# Cody

**Published:** 2015-08-06

**Authors:** Stephen M. Milner, James A. Fauerbach, Anne Hahn, Leigh Ann Price, Linda Ware, Kelly Krout, Elizabeth Panter, Nicole Kiehle, James Pfeiffer, Hien Nguyen, Geeta Sood, Kamal Dhanjani, Genevieve McKeon, Kevin Gerold

**Affiliations:** Johns Hopkins Burn Center, Johns Hopkins University School of Medicine, Baltimore MD

**Keywords:** burns, resuscitation, rehabilitation, psychosocial

## Abstract

Advances in burn management over the past 2 decades have resulted in improved survival and reduced morbidity. The treatment of a single patient following a 90% total body surface area injury illustrates the intensity of labour and coordinated hospital care required for such catastrophically injured patients. Data were extracted from the medical records and from personal recollections of the individual members of the multidisciplinary team as well as from the patient. The clinical course and management of complications are described chronologically as a series of overlapping phases from admission to discharge.

“I play with fire when I’m bored,” he said. “I would pour gasoline on the concrete floor of the basement and light it.” On February 2, 2011, around 11:30 pm, Cody's brother was awakened by screams as Cody, engulfed in flames, ran terrified upstairs.

His brother used towels to extinguish the flames and then drove Cody to a local hospital. The emergency department staff assessed the extent of his burns, intubated him to protect his airway from obstruction, and initiated fluid resuscitation (Fig 1). Cody was transferred to the Johns Hopkins Burn Center about 3 hours after injury. The staff estimated that he sustained burns to 90% of his body, sparing only his feet and perineum.

During the next 500 days, he would undergo 60 operations, receive 70,000 cm2 of cadaver allograft, and be transfused with 160 units of blood. The total cost of his acute hospitalization was approximately $2,000,000.

We chronicle the care of Cody's massive burn injury provided by a multi-disciplinary burn team delivered in a series of overlapping phases from the admission to discharge (Fig 2).

## RESUSCITATION (0-48 HOURS)

During the initial 48-hours of care, attention was directed to protecting Cody's airway, supporting his respirations, and preventing hypovolemic burn shock and organ failure.

### Airway and Respiratory Concerns

**Figure 1:**
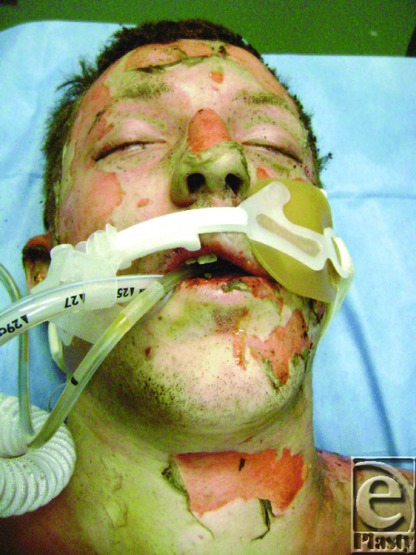
Cody on admission at 0200 on February 2, 2011.

**Figure 3:**
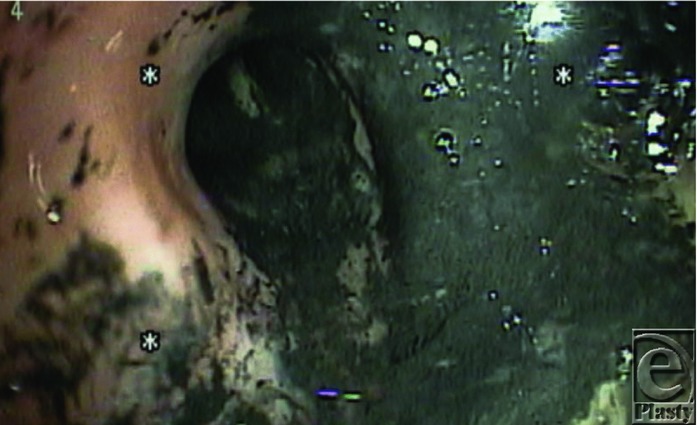
Bronchoscopy showing mild erythema with soot in the airways.

The deep burns to the face, scalp, hair, eyebrows and eyelashes, the history of burns occurring in a confined space, and the presence of carbonaceous sputum in the major airways all suggested the presence of inhalation injury (Fig 1). Fibre-optic bronchoscopy performed at the bedside confirmed the diagnosis (Fig 3).^1^ Endotracheal intubation from edema is obligatory during massive resuscitation, to guard against upper airway obstruction. Mechanical ventilation also permitted the administration of potent intravenous analgesic medications.

Inhalation of particulate and chemical constituents in smoke can give rise to a chemical tracheobronchitis that can extend into the alveoli, and is known to significantly increase mortality.^2^ 100% oxygen was administered over the first six hours to displace any carbon monoxide bound to hemoglobin and improve oxygen delivery.^3^ By 17 hours, his inspired oxygen concentration was reduced to 40%.

### Vascular Access

Venous access for fluid resuscitation was obtained by percutaneous placement of a triple lumen catheter into the right femoral vein; the groin providing the only suitable unburned site. A femoral arterial line was inserted for blood pressure measurement and blood gas analysis. Such catheters are potential sources of sepsis and were replaced weekly. The burn surgeons elected to excise and autograft the neck and clavicular areas to create additional sites suitable for central access.

### Fluid resuscitation

A large burn triggers a systemic inflammatory response and endothelial dysfunction, resulting in an increase in vascular permeability and changes in microvascular hydrostatic pressures. The imbalance of physical forces regulating fluid flux across the capillary causes a transfer of plasma-rich fluid from the vascular to the interstitial compartments.^4^ Failure to resuscitate patients undergoing these massive fluid shifts will lead to shock, decreased cardiac output and renal failure.

Traditionally, estimated fluid requirements following a large burn are calculated using mathematical formulae related to the weight of the patient and the size of the burn; the most popular in the United States being the Parkland formula.^5^ Actual fluid requirements are adjusted using urine output, blood pressure and other indirect measures of intravascular volume. The size of the burn as a percent of total body surface area (% TBSA) was estimated using a chart such as that described by Lund and Browder.^6^ Based on Cody’s% TBSA, his fluid requirements were estimated as 25 liters during the first 24 hours.

**Figure 4:**
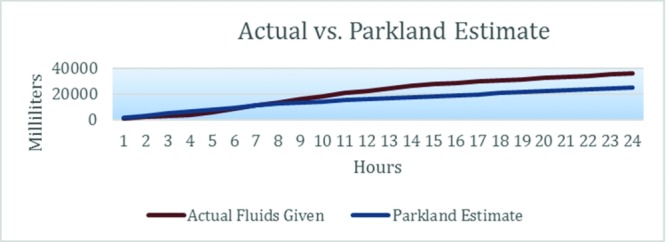
Cumulative fluids administered in first 24 hours.

It is not uncommon for patients with deep burns and inhalation injury for fluid needs to exceed calculated amounts. Reviewing the time course of fluid resuscitation, it was notable that Cody's early fluid requirements were just below Parkland estimates. His actual requirements, however, soon escalated well above his 24-hour Parkland Formula (Fig 4).

**Figure 5:**
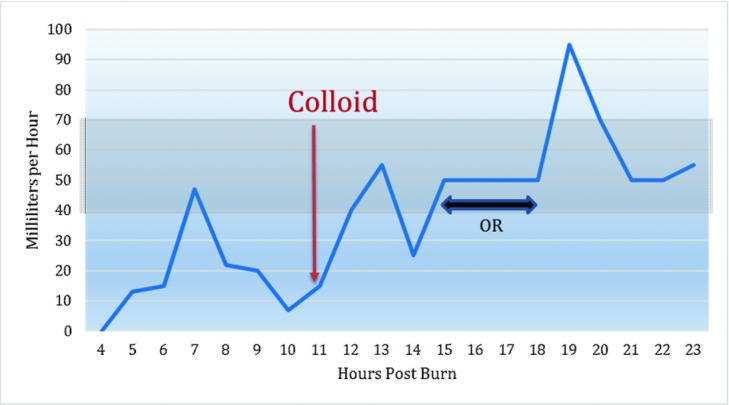
Urine output. OR indicates operating room.

To mitigate the fluid shifts, albumin was added to the resuscitation at 11 hours. The addition of colloid improved Cody's blood pressure and his urine output increased to the goal of over 0.5-1.0 ml/kg/hr. (Fig 5).

**Figure 6:**
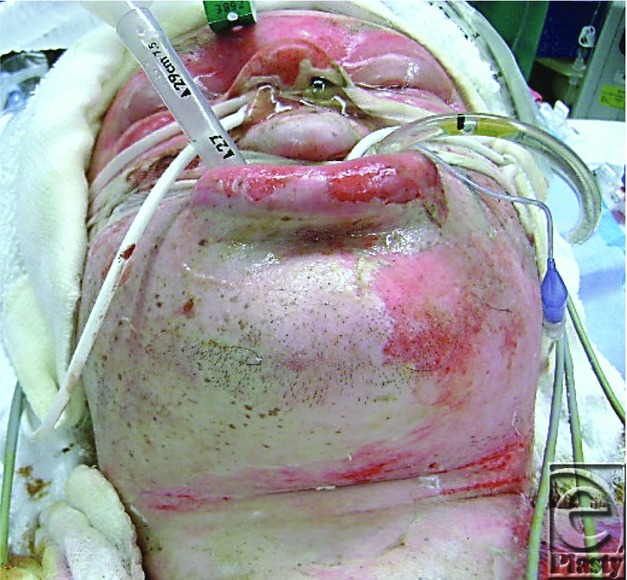
Facial oedema 12 hours after admission.

The effect of administering large volumes of intravenous fluids on Cody's appearance was notable as soon as 12 hours after admission, and the gross oedema to his head and neck caught Cody's brother completely off guard. He would report later that he was horrified by his brother's swollen features (Fig 6). This obligatory burn oedema, risked additional challenges to Cody's overall management. Excessive oedema can result in vascular compromise, upper airway obstruction and the conversion of viable deep second-degree burn to a non-viable full thickness injury. A careful, hour-to-hour assessment of fluid requirements is required to avoid the administration of excess resuscitative fluids, known as fluid creep.^7^

### Pulmonary complications

**Figure 7a:**
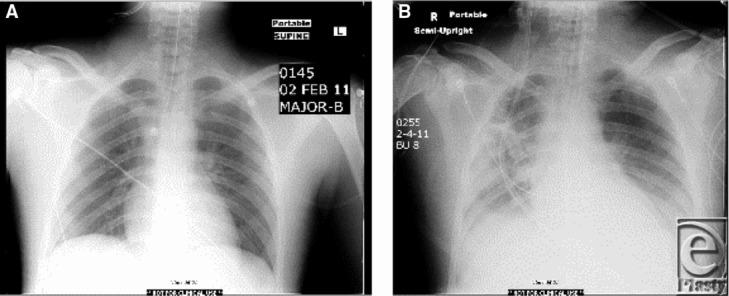
Cody's normal chest xray on admission. **7b.** Pulmonary edema day 2.

**Figure 8:**
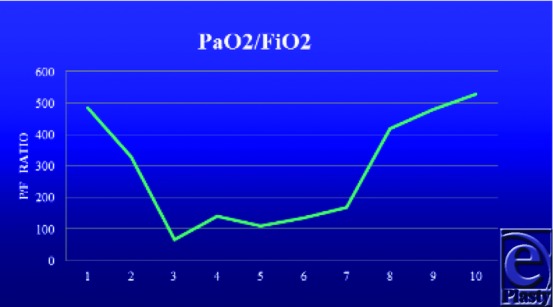
Fall in PaO_2_/FiO_2_ ratio.

By the end of day 2, Cody experienced worsening of his lung function attributed to acute lung injury from smoke inhalation, the systemic inflammatory response, and edema resulting from large volume resuscitation (Fig 7a and 7b). In addition, burns to his anterior thorax added to his decreased lung compliance. Due to concern for Adult Respiratory Distress Syndrome (ARDS) he was placed on a lung protective ventilator strategy with low tidal volume, and escharotomies of his chest were performed to increase chest wall compliance. This resulted in an improvement of gas exchange, and his FiO_2_ requirements were reduced from 1.0 to 0.40 (Fig 8).

It is estimated that up to 70% of patients with inhalation injury will develop ventilator-associated pneumonia.^8^ On day 6, Cody experienced worsening oxygenation, clinical signs of sepsis, and radiographic imaging consistent with pneumonia and ARDS. He was started on antibiotics and his oxygenation and clinical signs improved over the next few days. Six days later he underwent tracheostomy. He was eventually weaned from the ventilator, which was then reserved for surgery and the extensive dressing changes requiring propofol and narcotics for sedation and pain management.

### Social work

As Cody experienced profound metabolic disequilibrium, his family also experienced social and emotional instability. They were acutely confronted with a crisis situation for which they were not prepared, and lacked sufficient coping skills.^9^^,^^10^ To assist, the social worker began meeting with the family in the first 24-48 hours, which was organized around a caring but often absent mother. The structure was not reliable or stable, and the roles of the family not well defined. A major challenge for the social worker was to redirect Cody's mother's anxiety into more productive activities, and involved her participating in individual and support group meetings.

### Nutrition

Enteral feeding began immediately and was quickly advanced to goal in order to preserve gut mucosal integrity, prevent bacterial translocation and achieve nutritional goals during the burn related hypermetabolic phase, and to promote wound healing.^11^ This hypermetabolic response was exacerbated by fever, pain, burn eschar, multiple surgical procedures and infection, and would continue until all wounds were closed.^12^ Tube feedings consisted of 95 ml/hr. of a high calorie, protein dense tube feeding formula (Pivot 1.5). By 36 hours, Cody was receiving his estimated calorie and protein needs, 45 kcal/kg, and 2.5g/kg, respectively.

By 48 hours, Cody experienced a significant worsening in his pulmonary function (Figs 7a, 7b and 8). Because of initial concern for ARDS, his tube feeding formula was changed to Oxepa, which contains Omega 3 fatty acids, Eicosapentaenoic acid (EPA) and docosahexanoic acid (DHA). Both have been shown to inhibit eicosanoid production and modulate the inflammatory response in patients with acute lung injury.^13^ Tube feeding formula was changed back to Pivot when his lung function improved.

### Burn Compartment Syndrome

Within 2 hours of admission, Cody's hands and feet became mottled and cold, and the pulses were attenuated. Escharotomies were performed to release the compression caused by edema forming beneath the circumferential rigid burned tissue. These procedures were performed in the ICU at the bedside under intravenous conscious sedation. Incisions were made along the mid-lateral lines of the limbs using an electro-cautery. In the upper extremities, care was taken to avoid injury to the ulnar nerve behind the medial epicondyle, and the superficial radial nerve over the dorsum of the wrist. Dorsal escharotomies of the hands were made in the web spaces to decompress the intrinsic muscle compartments. Frequent vascular checks were continued using Doppler ultrasound with the intention to extend the escharotomy incisions if perfusion was inadequate.

### Rehabilitation

Cody was evaluated by occupational therapy and physical therapy on the day of admission.^14^ Their immediate goal was to preserve range of motion (ROM). This was complicated by burns which crossed almost every joint. After undergoing escharotomies and his wounds were dressed, Cody was positioned to prevent contracture formation, decrease edema, and maintain ROM. This included the fabrication of hand and knee extension splints and application of foot boots.

Neck contractures were prevented by positioning in neutral and avoiding using a pillow. Extremities were elevated and shoulders were positioned in abduction to prevent axillary contractures.

The rehabilitation phase would continue throughout the resuscitation phase and beyond. Positioning, splinting, and exercising all joints through ROM, actively if Cody was able to participate and passively if not. Treatment of both hands was limited to passive ROM to avoid tendon rupture.

### Abdominal escharotomies

**Figure 9:**
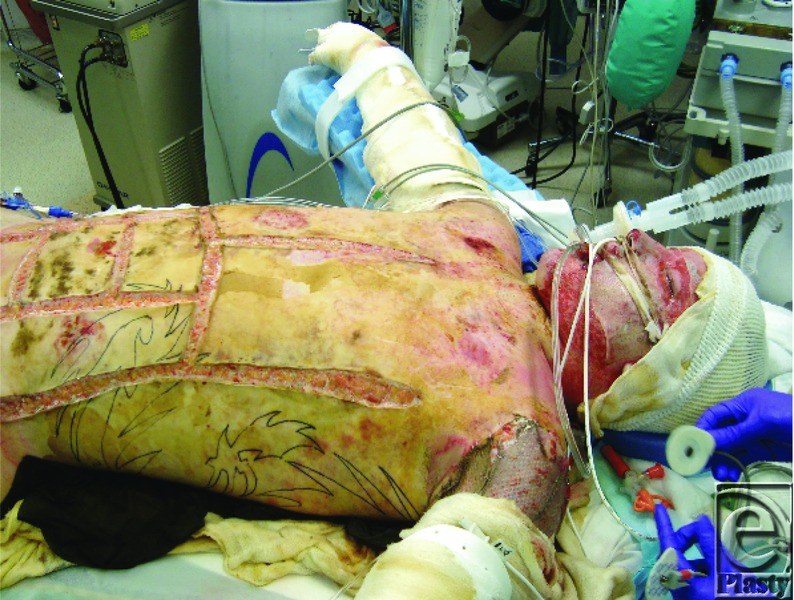
Chest and abdominal wall escharotomies.

A great concern during the initial burn resuscitation is the risk of intra-abdominal hypertension, which has an associated mortality in burn patients of over 70%.^15^ With the large amount of resuscitative fluids administered, Cody's abdomen became increasingly taut, his urine output dropped suddenly from 50 to 13 ml/hour, and his bladder pressure rose to 17 mm Hg. To reduce the intraabdominal pressure, escharotomies were performed across the anterior abdominal wall (Fig 9).

### Thermoregulation

Severe burns impair thermoregulation. Core temperatures below 35°C result in impaired blood clotting, platelet dysfunction and immune suppression. Ambient temperature of the ICU room and the operating room (OR) was maintained to prevent hypothermia, and wound care was provided beneath a radiant warmer.^16^

### Initial Wound Care

Following initial resuscitation, the burn nurses cleansed and debrided Cody's wounds, and dressed them with the topical antibacterial agent, 1% silver sulfadiazine cream. This topical cream has antimicrobial activity against a wide range of potential pathogens, including *Staphylococcus. aureus, Eschericia. coli, Klebsiella, Pseudomonas aeruginosa, Proteus,* the Enterobacteriaceae and *Candida albicans*. Systemic antibiotics were not used for prophylaxis as they do not penetrate the avascular eschar and increase risk of opportunistic infections.^17^

### Psychology

Common problems that develop in patients with severe burns include: pain, delirium, sleep disturbance, posttraumatic stress disorder, body image dissatisfaction, social discomfort and depression.^18^ Psychological burn-related issues are attended to as early as practicable. Even with intubated patients questions can be framed such that answers can be given in easily understood “yes” or “no” format (eg, thumbs up, eye movement).

Nurses and psychologists teach those patients with impaired verbal communication how to interact using nonverbal methods. For example patients can raise a finger or hand, move their head, or produce meaningful eye blinks. They may use subtle emotional clues such as by holding the examiners gaze. Movements of facial and eye muscles can express sadness, anger or happiness. Even while intubated, some patients are able to communicate by mouthing their words. However, owing to Cody's frequent need for deep sedation and recurrent episodes of critical illness, the psychologist was not able to work consistently with Cody in a therapeutic way until several months after his admission. Nonetheless, psycho-therapeutic interventions were provided intermittently during the earliest period with the conviction that, on a deep psychophysiological level, the brain and body systems might respond to, for example, relaxing and soothing tones.

The learned helplessness that started early continued throughout Cody's hospitalization.^19^ He also experienced periods of improved confidence and mood, alternating with profound hopelessness and depression associated with a passive death wish.

About one year after his injury, Cody manifested frequent episodes of trying to punch his care providers. Fortunately he was so completely deconditioned and his range of motion so limited that he did not pose a threat to the staff. Cody also went through periods when his rage and physical vitality were totally exhausted and he felt he could not go on living.^20^ During his time he told his care providers “Kill me!” With psychotherapy he was able to understand this was a non-helpful way of expressing his utterly complete exhaustion in the setting of seemingly endless, daily painful and demanding wound care and rehabilitation sessions. He acknowledged then, that he did not want to die but that his hopelessness and pain were overwhelming his coping resources. He was provided ongoing cognitive behavioural therapy, and renewed relaxation and mindfulness meditation assisted him with restoring physical and mental reserves.^21^^,^ 22^,^^23^

### Pain Management

Patients with significant burn injuries experience severe pain throughout their hospitalization. Managing Cody's pain was complex, required large doses of opioids, and was hindered by his increasing tolerance to narcotic agents. Efforts to address his constantly changing needs for analgesics along with adjunctive medications and non-pharmacological therapies helped Cody throughout his hospitalization and recovery, and permitted him to wean from narcotic analgesics safely over time.

He initially received morphine and midazolam infusions, with rates that reached as high as 9 mg/h and 6 mg/h, respectively. Infusions were titrated to his pain score goal and additional medications were administered during dressing changes; he received fentanyl 400 mcg, hydromorphone 2 mg, and propofol by infusion for this procedure. Eventually his regimen included methadone 30 mg every 8 hours, oxycodone 15mg every 4 hours as needed, hydromorphone 12 mg IV for dressing changes, and duloxetine 60 mg every 12 hours.^24^ In his tenth month it was felt that he had developed opiate induced hyperalgesia.^25^ A ketamine infusion was initiated in an effort to reset his narcotic threshold and reduce his need for narcotic analgesics.^26^^,^^27^ This infusion was continued for 5 days at a low dose of 0.08mg/kg/hour. Multi-modal pain management strategy is required for burn patients especially in cases of severe burns.^28^

## BURN EXCISION (12hrs–14 days)

The standard of care for major full thickness burns is early staged excision.^29^ The principle is to remove all eschar promptly and resurface the wound with available autograft, temporary biological dressing or skin substitute until autologous donor site becomes available. Since dead tissue is responsible for infection, the cytokine storm, the systemic inflammatory response and the metabolic effects, the patient's only chance for survival was for the eschar to be removed quickly.^30^

Surgical excision of Cody's burn wounds began at 12 hours following admission. Since his upper extremities continued to show evidence of vascular compromise the wounds were excised shortly before his 21^st^ birthday.

Fascial excision, removal of skin and subcutaneous tissue to muscle fascia, was preferred to the more conservative tangential excision, since fascia provides the optimum surface to receive a skin graft and the risk of graft failure is reduced. This is especially important when donor sites are limited. Secondly, this type of excision is performed using an electro cautery and minimizes blood loss. Using tourniquets and topical epinephrine the excision of the upper extremities was performed with blood loss of only 200 ml.^31^ In order to prevent hypothermia, the room was maintained at 85°C which maintained the patient's temperature throughout the entire case to within 1.5°C of his starting temperature of 37.2°C.

**Figure 10:**
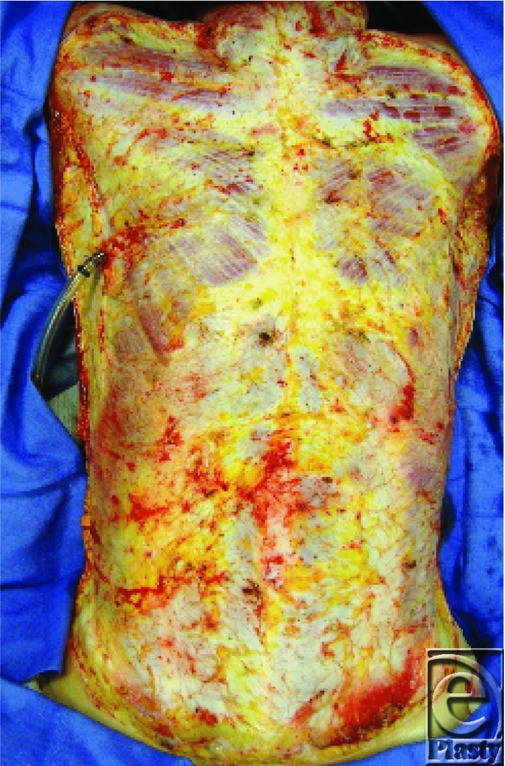
Fascial excision of chest post burn day 3.

A second excisional procedure was performed at post burn day 3 with excision of the abdomen and chest (Fig 10). The small amount of unburned skin available was harvested and grafted to burned areas over the neck and clavicle. Once healed in about a week to ten days, these areas would permit the use of these sites for tracheostomy and for central vascular access.

The second surgery also included harvesting a full thickness skin biopsy of unburned skin. This specimen would be sent to Genzyme in Boston, MA in order to grow cultured epithelial cells. It would take three weeks before the resulting cultured cells would be available for grafting.

**Figure 11:**
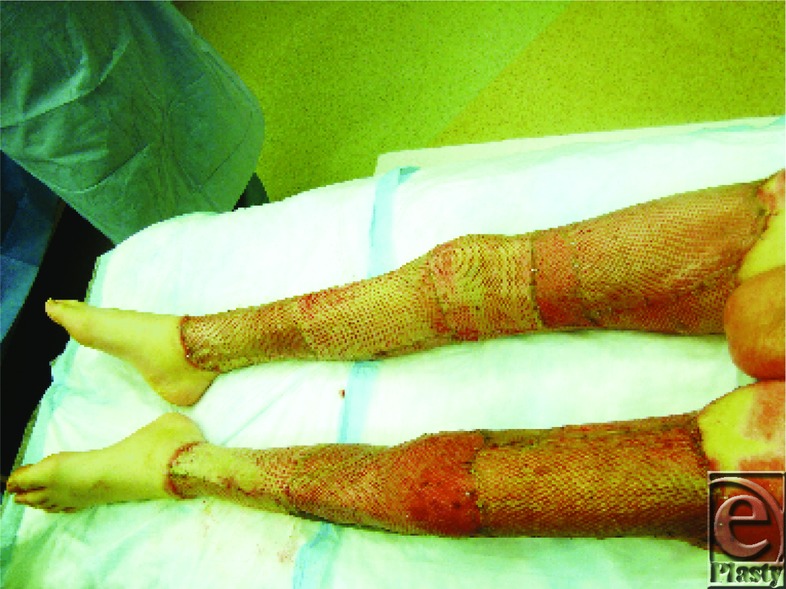
Lower extremities covered with allograft.

The lower extremities were excised and resurfaced temporarily with human cadaver allograft on post burn day 5 (Fig 11).

The excision of the back and dorsum of the hands on day 8 completed the full excision of the burn wound. On day 10 the patient returned to the OR for k-wire pinning of the fingers to protect the proximal interphalangeal (PIP) joints. At the same time, a tracheostomy was performed using a semi-open, percutaneous technique.^32^ Splints were applied in the OR after the completion of surgery to prevent movement of joints and shearing of grafts.

The face was treated conservatively by gentle washing with mild soap and application of bacitracin ointment. By keeping the wound moist and free of infection, viable cells in the dermis around the skin adnexa were preserved to encourage skin regeneration.

**Figure 12:**
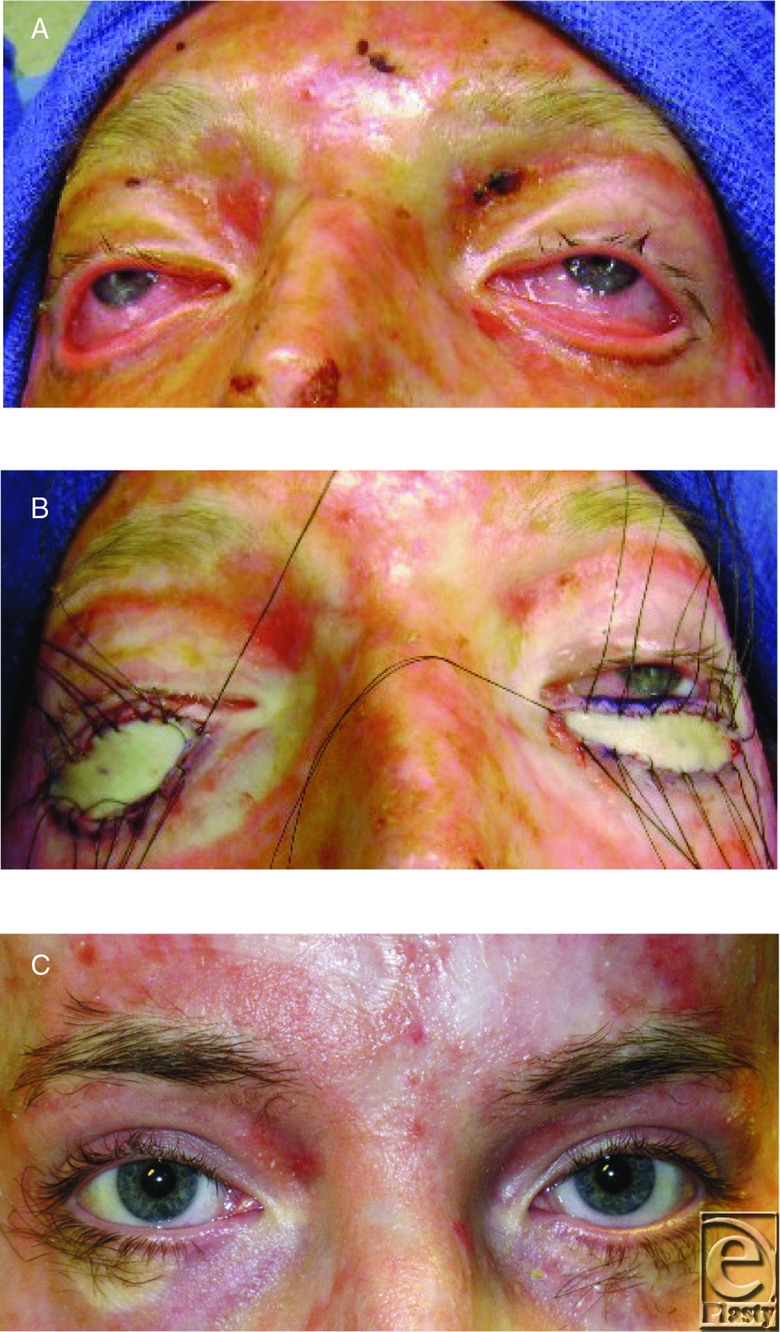
(a) Ectropion of lower eyelids. (b) Release of lower lid ectropions and full thickness skin-grafts. (c) Ectropions corrected, restoring protection of the cornea, a year after a surgery.

Reconstruction of the face began when there were functional problems. Early correction of ectropions was were performed to prevent desiccation of the eyes. Lower eyelid contractures were released and grafted using full thickness skin grafts from the groin (Figures 12a-12c).^33^

Infections are an inevitable consequence of large open wounds, which become colonized with potential pathogens. Studies demonstrate that patients undergoing surgery or wound manipulation become bacteremic 30-50% of the time.^34^ It was difficult to prevent local wound infections from spreading to or “seeding” the bloodstream and the central venous lines. To reduce wound infections, strict aseptic techniques are instituted, along with surgical debridement and topical antibacterial agents. Wound infections precipitated allograft failure and line contamination.

**Figure 13:**
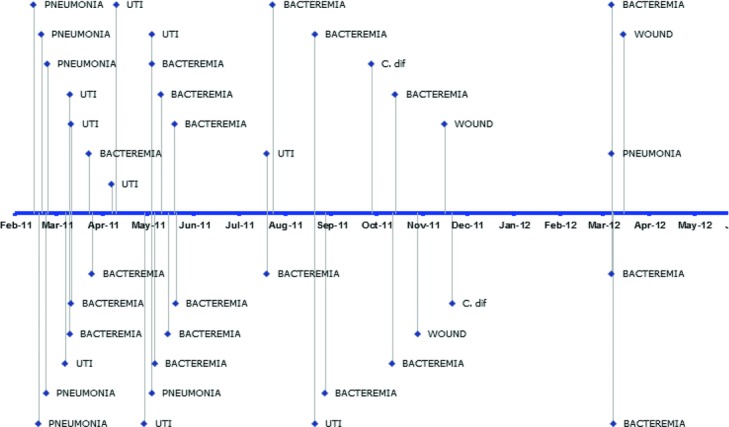
Infectious disease recurrence during a 500-day intensive care stay. The UTI indicates urinary tract infection; C. diff, *Clostridium difficile* infection.

Cody experienced 15 episodes of bacteremia 6 of which were attributable to central line-associated infections (Fig 13). Catheter-associated blood stream infections are minimized or prevented by adopting a policy of scheduled line changes.

Bacterial flora in his sputum, urine, and wounds were monitored carefully to distinguish between colonization and active infection. 18 different organisms were grown from wounds, blood, sputum and urine. Systemic antibiotics were administered only when clinically indicated for infection. Depsite efforts to limit the use of antibiotics, Cody received sytemic antibiotics for 212 days (41%) of his 513 day hospitalization; mainly for blood stream infections. There were also 4 episodes of treated symptomatic urinary tract infection (UTI) and 2 episodes of *Clostridium difficile* infection.

## RESURFACING PHASE (1 WEEK–1 YEAR)

**Figure 14:**
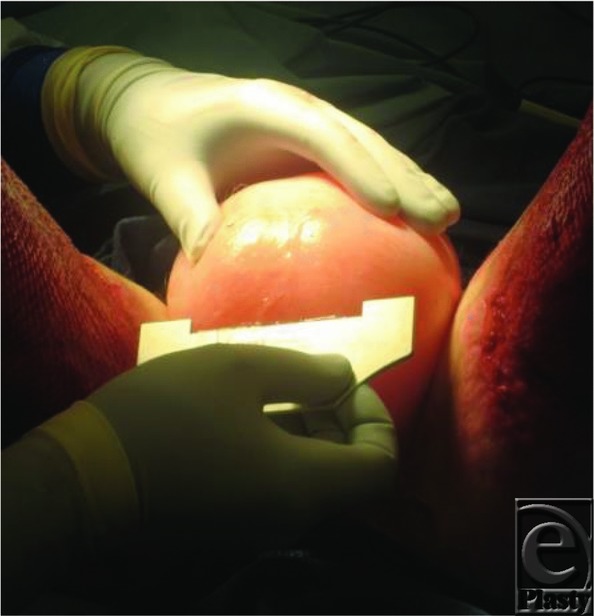
Harvesting skin from the scrotum.

Cody's survival would depend ultimately on being able to cover him with his own skin. With so little unburned skin available, however, it became necessary to harvest from unusual donor sites (Fig 14). To facilitate harvesting donor skin from the scrotum, it was first expanded by injection of saline to create, in effect, a large hydrocele.

**Figure 15:**
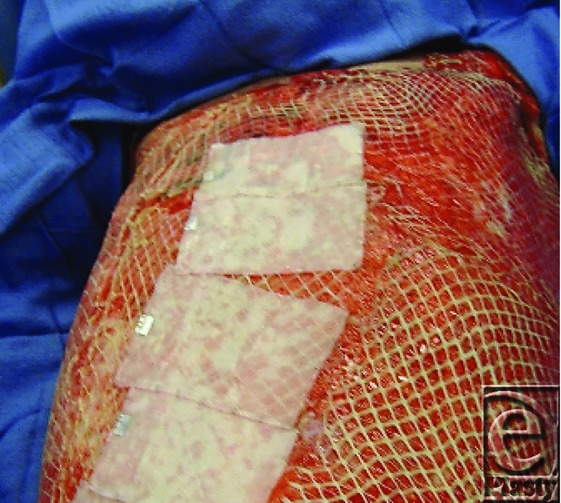
CEA place over 4:1 meshed autograft.

The culture and transplantation of keratinocytes was a major advance in the treatment of large burns.^35^ Grown from a harvested specimen of the patient's unburned skin early after admission, epithelial regeneration under laboratory conditions produced quantities of epithelial tissue sufficient for grafting in three weeks (Fig 15).

Our early results using cultured epithelial autograft (CEA) have been disappointing. Since cultured epithelial autograft (CEA) lacks a dermal component, the CEA is fragile and take is often poor.^36^ To overcome these problems the CEA was placed over widely expanded meshed (4:1) autologous split-thickness skin (Fig 15).

### Nursing

The CEA was treated with gentamycin spray and left open to air. One nurse described the first dressing change for the CEA as follows:

“We don't have the luxury of taking the patient to the OR, where the patient is asleep, and there are plenty of assistants. You’re wearing a hat and three pairs of gloves. The temperature is 110°F and the heat shield is burning your head. The CEA rep is getting mad because you’re sweating on the graft and it's very uncomfortable, especially when you’re pregnant. First you have to remove the backing of the CEA without damaging the cells themselves and that's a lot of money. One hand picks up the forceps, the other hand wets the backing and the third hand is giving pain medicine and turning off the ventilator, which is alarming because he's coughing”

**Figure 16:**
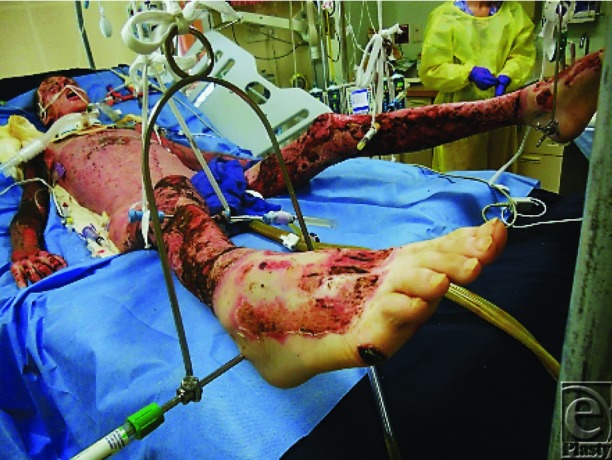
Legs suspended for 6 weeks using Steinman pins to decrease shear on CEA.

**Figure 17a-17c:**
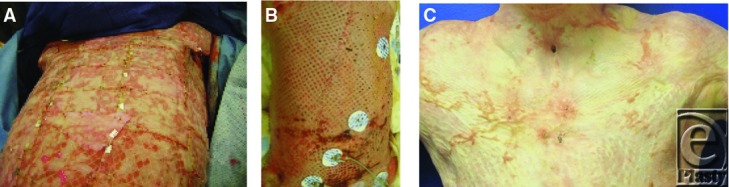
Progression of healing of anterior trunk.

The initial dressing change took 6 hours. Changing the sheets in Cody's bed at this time had to be carried out without turning him, with his legs in traction. It took 5 people (Fig 16). Within a couple of weeks Cody's anterior trunk healed well (Fig 17a–17c).

### Psychology

**Figure 18:**
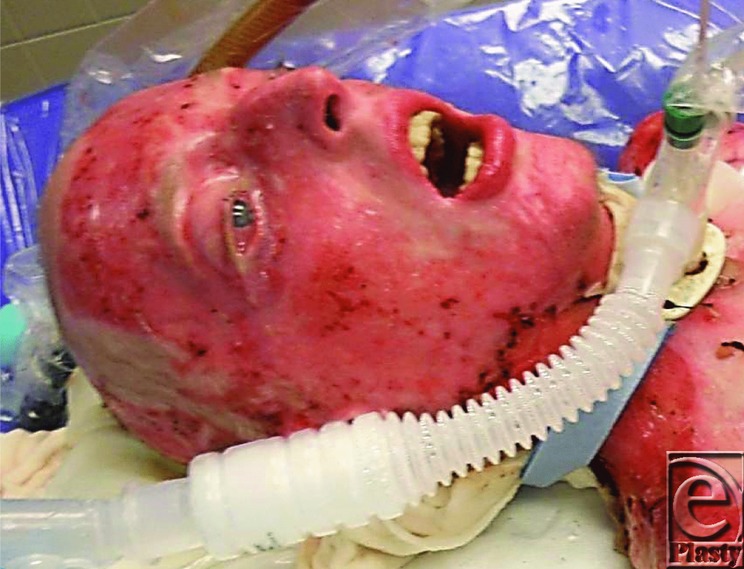
Cody during therapy.

As Cody's wounds healed and he became able to participate in his care, he developed further behavioural problems. Physical and verbal abuse became his only form of retaliation. He told one of the pregnant nurses he was going to burn her baby, he was going to get his boys to kill her and he would turn his tracheostomy and cough towards her. He would say things such as “This line belongs to me. I have rights and if you don't take it out I’ll sue you.” Questioning him afterwards about his behaviour he said he just lay there in isolation and felt people were coming in for an hour at a time to torture him (Fig 18).

One of the first concerns Cody expressed when he actually started treatment with the psychologist was about his procedural pain which he described as excruciating, unbearable and exhausting.^37^ He had been undergoing twice-daily dressing changes and once-daily rehabilitation sessions since admission. For the times when he was sufficiently awake, these sessions, which he reported as uncontrollable, unavoidable and acutely painful, had resulted in him developing a learned helplessness whereby he saw the problems and solutions as external to himself so he had no control, global so that his entire day was consumed in thinking about it, and stable in that he believed there was nothing he could do to improve the situation. He developed understandable but unhelpful ways of thinking, feeling and behaving towards these painful episodes. Cody would become highly anxious prior to dressing changes and he became hypervigilant for nuanced signals of increased pain during them. As he waited for his procedures, he would recall past episodes of unbearable pain and he would anticipate that the upcoming procedure would likely be even more excruciating. These “catastrophising” thoughts are strongly reinforced by each instance of intense pain. Even more problematic, the catastrophising actually increases the sensitivity of pain receptors intensifying subsequent pain.^38^

The cognitive-behavioral model of pain management has been proven in many pain settings to reduce pain and improve mood.^39^ This is accomplished by challenging thoughts (eg, examining evidence; providing more useful ways of thinking that empower the patient), teaching ways to relax the anxious tension felt throughout the body (eg,, relaxation, breathing meditation, guided imagery), and present sensory focusing (eg, cooperative communication with team). Cody, for example, was provided guided imagery and breathing retraining to induce relaxation. His ability to benefit from this sort of input was observed through heart rate reductions and, in the slight lessening of facial tension, shoulders and hands tension, and a loosening of the body allowing the torso and head to relax further into the mattress and pillow.

### Feeding

**Figure 19:**
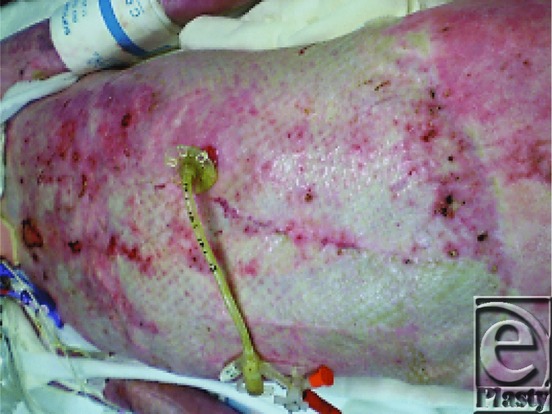
Percutaneous endoscopic gastrostomy tube in situ.

Two months after his injury, Cody had progressed to where he was able to swallow thickened liquids, but not solid food. In order to provide his nutritional needs long-term, his nasogastric feeding tube was replaced with a percutaneous endoscopic gastrostomy, (PEG) (Fig 19). While this is a standard procedure used for long-term nutritional support, the absence of subcutaneous fat and the need to perform the PEG through grafted skin resulted in erosion of the gastrocutaneous tract. This resulted in significant leakage of tube feeding. Interventional radiology upsized the catheter to seal the leak, but the tract widened further. Advancing the PEG catheter into the jejunum also (PEG-J) failed to reduce the leak.

Erosion of the fistula continued to where food and medications were escaping around his PEG. The team considered putting the stomach at rest and permitting the fistula to heal by placing Cody on total parenteral nutrition. Instead he was fed using a distally placed naso-jejunal tube (Tiger 2 Tube). After about 6 weeks the site had healed sufficiently to replace a much smaller PEG. Over the next three months Cody was allowed to resume eating and we were able change from continuous to cycled tube feeding. Cody's PEG tube was removed prior to discharge to rehabilitation. His fistula continued to drain and this persisted as one of his main complaints. Two years after hospital discharge and following several procedures performed to close the fistula, the PEG site finally healed and is no longer leaking.

### Speech/swallowing

Prolonged intubation and tracheostomy leads to glottic edema, impaired sensation and abnormal function in the mechanics of swallowing. This can contribute to aspiration of food and liquid into the airways and pneumonia. Once removed from the ventilator, Cody would have to pass a swallowing evaluation before being permitted to eat or drink. In advance of this test, a Passy-Muir Valve was attached to Cody's tracheostomy tube to help re-establish airflow and increase sensation by directing air through the pharynx. This allowed closure of the vocal cords permitting him to speak with the tracheostomy in place and helped to restore normal pressures associated with swallowing.

**Figure 20:**
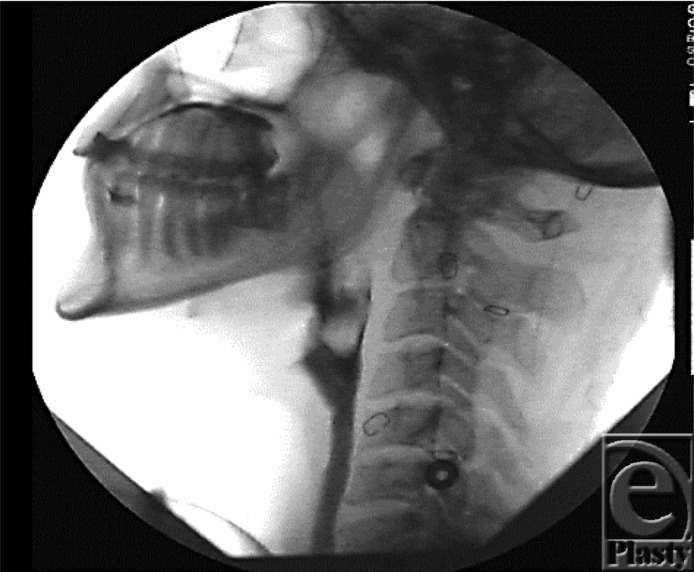
Video fluoroscopic examination showing no tracheal aspiration.

Conducting a swallowing evaluation six weeks after Cody's injury was a clinical challenge. Cody fatigued easily, had difficulty sitting upright, and experienced arterial oxygen desaturation when off oxygen. A flexible endoscopic evaluation of swallowing (FEES) was performed avoiding transfer to the radiology department. Fear of pain necessitated the test be performed with sedation. After the first of three swallow studies during his admission, Cody was cleared for nectar thickened liquids. Five months into the Burn Intensive Care Unit admission, Cody graduated to a regular diet with thin liquids after demonstrating functional swallowing on a video fluoroscopy (Fig 20).

While Cody was able to consume a regular diet, the limited flexion at his elbows made it impossible for him to feed himself without assistive devices. Occupational therapy was able to fabricate long-handled feeding utensils and an extended straw that enabled Cody to feed himself and provided a degree of autonomy.

Then there were other members of the burn team actively involved in Cody's treatment and care. The Chaplain would read to him for hours. He told me one of Cody's favourites was the story of Elijah. “It's ironic,” he said. “It's about fire.”

We also consider the family as part of the team, for ultimately the patient will be discharged to their care. Families cannot be expected to transform themselves from dysfunctional behaviours to consistent rational behaviours. However. Through consistent support, information, education and empathy, family dysfunction can be lessened.

### Final Wound Healing

As the CEA did not heal well on Cody's the back, and to overcome the limitations of limited donor sites, we resorted to the Alexander technique.^40^ Here, widely expanded meshed (4:1) autologous split-thickness skin grafts were overlaid with unexpanded (2:1) meshed allograft split skin. This “sandwich technique” promoted rapid epithelialization of the interstices of the autograft and shedding of the allograft before inducing acute rejection.

**Figure 21:**
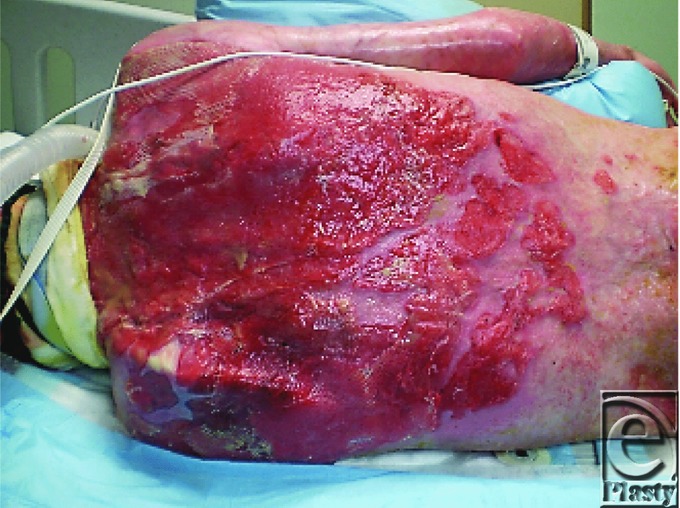
Delayed healing in lower back.

**Figure 22:**
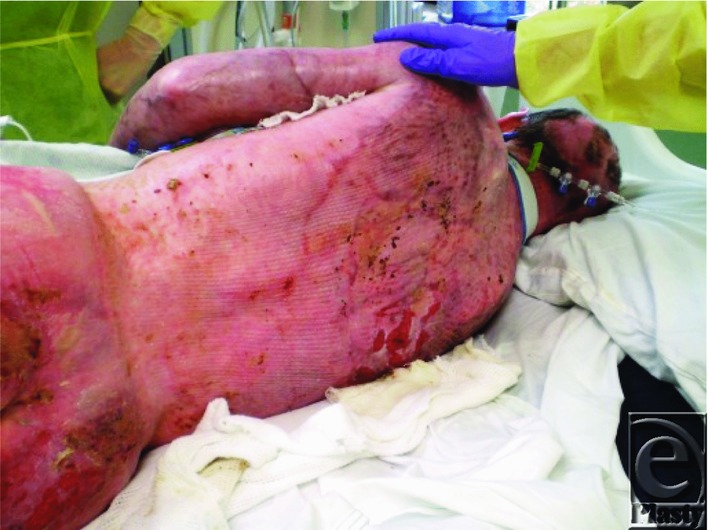
Back finally healed.

This worked better in some areas, such as the lower back (Fig 21) but not in other areas (e.g., where the body pressed against the bed). With time and with the use huge negative pressure dressings and the nursing skills the back wounds healed (figure 22).

## REHABILITATION AND RECONSTRUCTION

**Figure 23:**
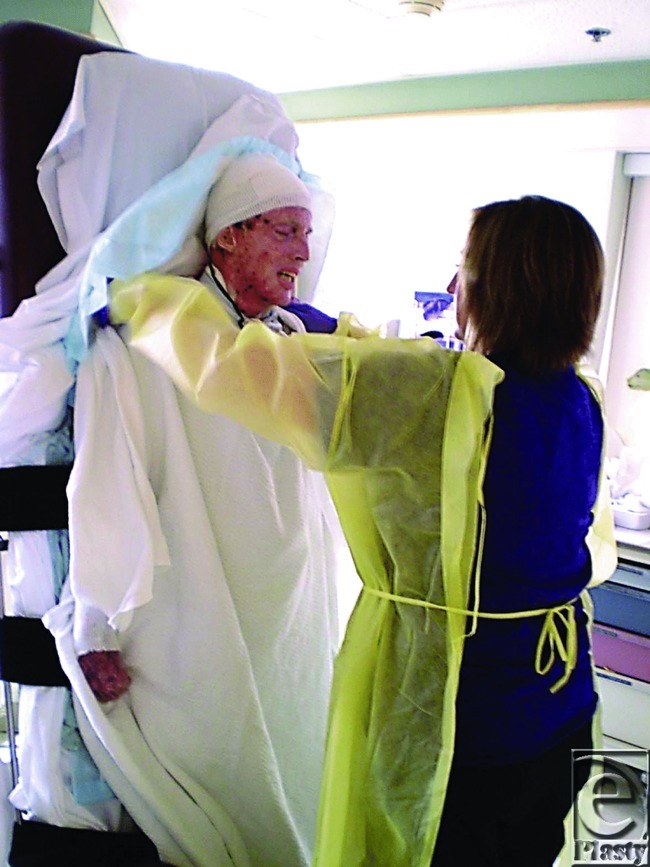
Cody rendered vertical for the first time since he was injured with the help of the tilt table.

By the end of May 2011, Cody for the first time was able to tolerate sitting up for 4 hours. Three therapists were needed to lift him from the bed into a recliner, extra care being taken to not shear the fragile skin on his back and buttocks. After a week of sitting up he attempted standing with use of a tilt table (Fig 23). The next few months were challenging with slow improvement in mobility due to pain in his feet and wounds, further surgeries and the patient's frustration and refusal to work with therapists. Cody sometimes became so distressed that he would make himself vomit at the time of his therapy sessions. He would comment, “It's over. Just let me die. I can't do this anymore.” However, with support from therapists, he stood for the first time in July and was sitting up playing computer gaming systems by August.

Further surgeries and poor ankle motion slowed his ability to walk. Serial casting, dynasplints, standing frame and botox injections were used to correct the ankle contractures. The pain, distress and hopelessness recurred at times. For example, on one occasion, after standing in the standing frame for 30 minutes, he told the therapy staff “if I die it's because of you.”

Hand function improved with serial casting and dynamic splinting and in July Cody was able to stand in parallel bars and grip the rails. At the end of the month he was able to feed himself, drink and use a computer.

**Figure 24:**
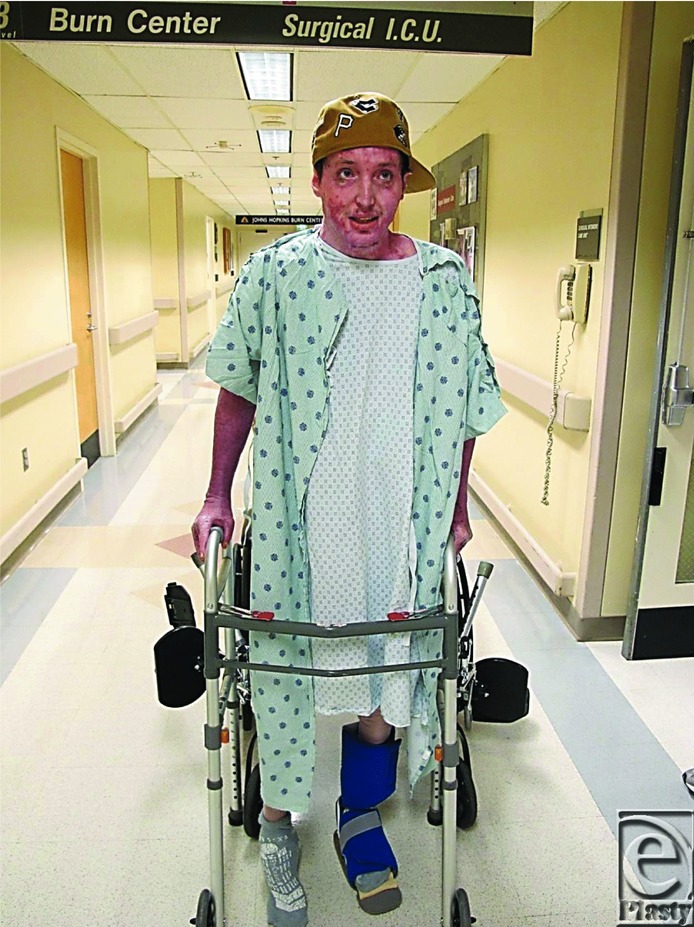
Walking with the help of a platform walker.

**Figure 25:**
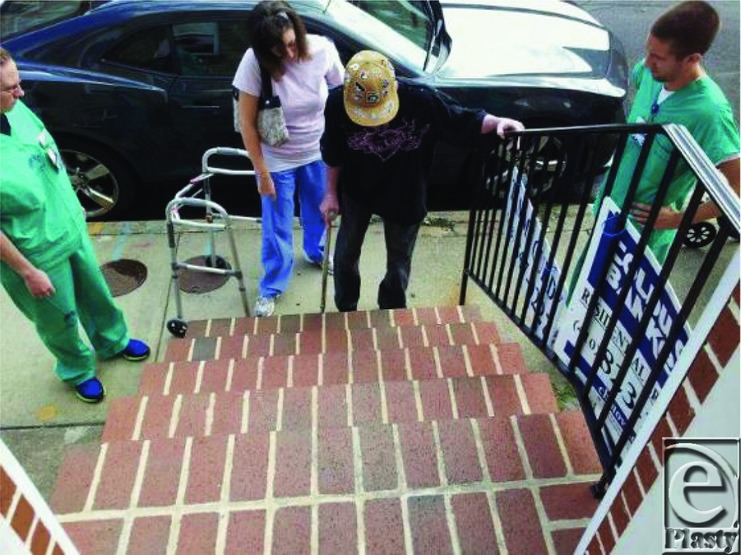
Walking out of the hospital.

In December, Cody was able to walk short distances using the platform walker (Fig 24), his progress again impeded by further surgeries and extended bed rest. He began to recognize his progress and the pride of his accomplishments began to show in increased effort during rehabilitation. Cody was able to leave the hospital 18 months after his injury (Figure 25).

### Discharge

Cody's mother did find positive resolutions to her son's injury. She changed jobs to one with better pay and working hours, which allowed her more time to devote to her son's recovery. She moved to a new home that provided easier accessibility for Cody.

Having survived and recovered from his severe burns, Cody's functional and cosmetic concerns will be addressed. An inventory of Cody's wish list included his correcting his alopecia, a contour deformity of his upper thighs from his fascial excision, and a persistent tracheocutaneous fistula. Cody also has neck contractures and deformities of his ears. Reconstruction is planned for the future.

Surprisingly one of his main problems was an equinovarus deformity of his feet following uncontrolled suspension of his legs during the healing of the CEA. Serial casting and injection of phenol into the gastrocnemius and tibialis posterior failed. Osteotomies and tendon lengthening procedures, however, have provided a near normal range of ankle motion and restored his ability to walk.

**Figure 26:**
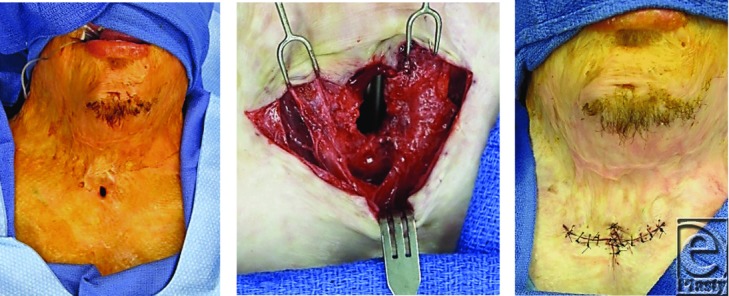
Persistent tracheal fistula **B.** Layered local flaps **C.** Wound closure.

A particular concern to Cody was a persistent trachocutaneous fistula resulting from incomplete closure of his tracheostomy. The tracheostomy was left in place until he was removed from the ventilator, had passed his swallowing evaluation, and no longer required acute surgery or complex wound care. Closure of the persistent tracheocutaneous fistula was accomplished using layered local flaps (Fig 26).

### Closure of Persistent Gastrocutaneous Fistula

Chronic use of the PEG tube led to the persistence of a gastrocutaneous fistula. These fistulas normally close 48 to 72 hours after the PEG is removed. Application of fibrin glue and antacids gave temporary relief until ultimately the fistulous tract was mechanically de-epithelialized and closed using vertical mattress sutures. He still reports a very active lifestyle without any drainage from the prior G-tube site.

### Social Reintegration

**Figure 27:**
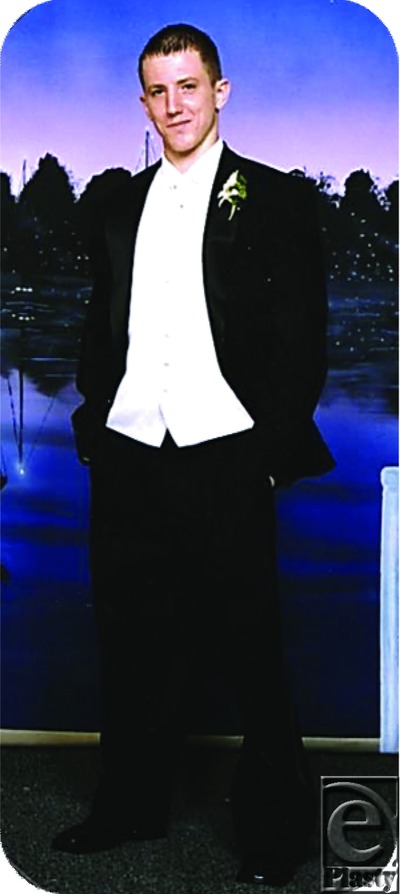
Cody at his high school prom.

Deep down Cody still suffers from social discomfort and depression. His emotional responses are a reaction to seeing his disfiguring scars, as well as from his own evaluation.^41^ Self-stigmatization is a form of rejection based on actual or perceived characteristics that one finds unacceptable because they conflict so greatly with one's preferred or remembered appearance and function.^42^ He was highly critical, self-demeaning and disgusted with his own scarred body. He remembered himself as the high school athlete and member of the National Honor Society. Throughout his stay he had a crown beneath his bed; a reminder of his time as the Prom King at his high school's Senior Prom (Fig 27). What he now perceived in the mirror and how he now graded his performance even with simple, everyday tasks, were challenges with which he was inadequately prepared to manage healthfully as a young man from an over-stressed, poorly organized family, and a neighbourhood of threat, crime and drugs.

Indeed it is hard to believe that with the complete cadre of team services given daily attention to Cody and his survival, and intermittent applications of cognitive behavioural therapy, the man whose journey through hell and back, is the same person who walked onto the ward to give us a plaque to say “thank you.” He said at the time he wanted to be an occupational therapist.

**Figure 28:**
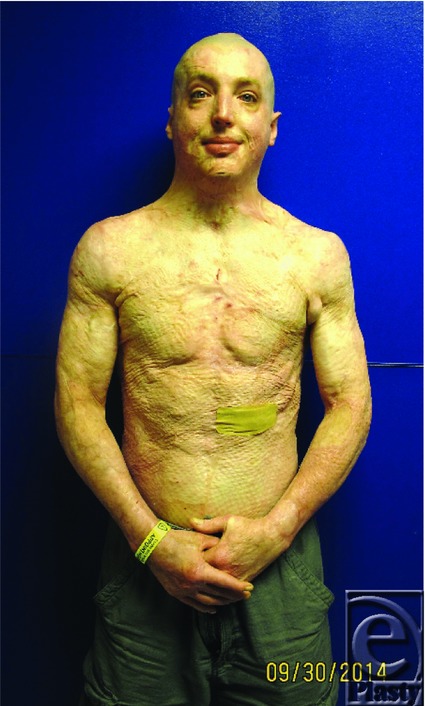
Cody's surgical and psychosocial progress to date.

Cody looks like he's coping well as shown by his expression and posture in Figure 28. I asked Cody if he recalled the intensity of his initial pain when he had just been burned. He said it was definitely a ten out of ten. Then he paused and said “but you know I was never sure what that meant. It's a bit like how you look at girls. Is it a Baltimore ten, or a Miami ten?”

We are thankful to Cody for his collaboration with this manuscript and have been privileged to observe his courage during recovery.

## Figures and Tables

**Figure 2:**
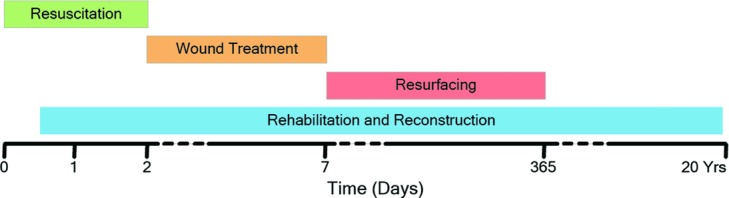
Phases of treatment.
